# Addition of Bamboo Charcoal to Selenium (Se)-Rich Feed Improves Growth and Antioxidant Capacity of Blunt Snout Bream (*Megalobrama amblycephala*)

**DOI:** 10.3390/ani11092585

**Published:** 2021-09-03

**Authors:** Fang Jiang, Yan Lin, Linghong Miao, Jingyuan Hao

**Affiliations:** 1 Key Laboratory for Genetic Breeding of Aquatic Animals and Aquaculture Biology, Freshwater Fisheries Research Centre (FFRC), Chinese Academy of Fishery Sciences (CAFS), Wuxi 214081, China; bf19972008@126.com (F.J.); liny@ffrc.cn (Y.L.); 2 Wuxi Fisheries College, Nanjing Agricultural University, Wuxi 214081, China; heiyanjing1984@163.com

**Keywords:** *Megalobrama amblycephala*, bamboo charcoal, selenium, growth performance, antioxidant ability, Nrf2/Keap1/NF-κB

## Abstract

**Simple Summary:**

In our previous studies, we determined that juvenile blunt snout bream (*Megalobrama amblycephala*) require a selenium (Se) dietary intake of 0.958–0.959 mg/kg based on the quadratic fitting method. Nevertheless, growth rates were significantly affected, and the expression of anti-inflammatory factors was inhibited when the feed contained excessively high Se concentrations. Given its activity as an insoluble carrier, bamboo charcoal can be used as a dietary supplement in fish feed to absorb and slowly release excess nutrients. However, these hypotheses have not been evaluated in diets containing excess Se. The present study determined that 2–3 g/kg bamboo charcoal inclusion in Se-rich feed could improve the growth performance of blunt snout bream. Dietary bamboo charcoal supplementation mobilized the antioxidant system and inhibited the inflammatory response by activating Nrf2-Keap1 signaling while suppressing NF-κB signaling.

**Abstract:**

The ability of bamboo charcoal to reduce the negative effects of high dietary selenium (Se) concentrations was assessed by feeding juvenile blunt snout bream (*Megalobrama amblycephala*) one of five Se-rich diets (1.5 mg/kg Se; 36% protein, 8.7% lipid) containing graded levels (0–4 g/kg) of bamboo charcoal powder for eight weeks. There were four tanks (350 L) of fish (initial weight 16.0 ± 0.5 g) for each treatment, and the fish were fed to satiation four times each day. At the end of the feeding trial, all of the fish from each tank were weighed to calculate the growth performance. Blood samples were firstly obtained to collect plasma for the biochemical indexes determination. Liver tissues were then collected to determine the antioxidant enzyme activities and gene expression. Dorsal muscles were also collected to determine the nutrient composition. The results show that when the bamboo charcoal content in the Se-rich feed ranged between 0 and 3 g/kg, the weight growth rate (WGR) and specific growth rate (SGR) values increased with the higher dietary bamboo charcoal content, and the maximum WGR and SGR values were achieved when the bamboo charcoal content in the Se-rich feed was 2–3 g/kg (*p* < 0.05). The Se content in muscle tissues decreased significantly with the increased bamboo charcoal content (*p* < 0.05) in the Se-rich feed, which ranged from 0 to 4 g/kg. When the bamboo charcoal content in the Se-rich feed was 2–3 g/kg, the levels of glucose (GLU) and albumin (ALB) in plasma reached a maximum (*p* < 0.05), whereas the level of alkaline phosphatase (ALP) reached a minimum (*p* < 0.05). Additionally, the activities of catalase (CAT), total superoxide dismutase (T-SOD), total antioxidative capacity (T-AOC), and glutathione peroxidase (GSH-Px) were significantly enhanced (*p* < 0.05) when the bamboo charcoal content was 3 g/kg. In contrast, the malondialdehyde (MDA) level increased sharply when the bamboo charcoal content in the Se-rich feed was 1 g/kg, compared to the control group and the groups supplemented with 2–3 g/kg bamboo charcoal (*p* < 0.05). Regarding mRNA-level gene expression, the results show that dietary supplementation with 0 to 3 g/kg of bamboo charcoal increased the expression of *keap1* and *nrf2*, whereas *nfkb* expression was inhibited (*p* < 0.05). The mRNA expression of the antioxidant enzymes *cat*, *gpx*, and *mn-sod* was consistently enhanced in the group fed with the 3 g/kg bamboo charcoal diet (*p* < 0.05). The expression of the pro-inflammatory cytokines *tnfα* and *tgfβ* was inhibited in the groups supplemented with 2–3 g/kg bamboo charcoal, whereas the expression of anti-inflammatory cytokines (*il10*) increased in the bamboo charcoal supplementation groups compared to the control group (*p* < 0.05). Generally, supplementation with 2–3 g/kg of bamboo charcoal in Se-rich feed improved the growth performance, physiological status, and antioxidant enzyme activities of blunt snout bream. Moreover, bamboo charcoal supplementation in Se-rich diets stimulated the antioxidant system and inhibited the inflammatory response by activating Nrf2-Keap1 and suppressing NF-κB.

## 1. Introduction

Selenium (Se) is an essential trace element that is widely thought to support oxidative stress tolerance in fish. Although the tolerance range of Se in biological systems is very narrow [1], the Se requirements of different fish may vary greatly not only in a species-dependent manner but also depending on the molecular form of Se (e.g., organic, inorganic, and nano-forms) [2,3,4]. Other factors that influence Se requirements include fish size, health status, dietary formulation, experimental conditions, and water quality [5]. Previous studies have indicated that the presence of appropriate Se contents in feed can promote fish growth, whereas excessive Se concentrations may inhibit growth [6,7,8]. Specifically, excessively high dietary Se concentrations lead to oxidative stress and an increased hemolysis rate, resulting in reproductive failure, tissue damage, and organ malformation [9,10,11]. Lee et al. [12] utilized Se methionine as a Se source and described that the growth performance, survival rate, and nonspecific immunity of Nile tilapia (*Oreochromis niloticus*) were significantly reduced when the Se intake was more than 2.06 mg Se/kg diet.

Bamboo charcoal (i.e., an activated carbon material) is made from the thick stems of bamboo through dry distillation. Importantly, this material can adsorb a variety of molecules and contains a complex network of pores of different shapes and sizes [13]. Due to the unique structure of the micropores in the bamboo stem, bamboo charcoal is thought to possess a higher adsorption capacity. For instance, this material has been used to adsorb ammonia nitrogen in water and decrease heavy metal intake [14]. Given its activity as an insoluble carrier, bamboo charcoal can be used as a dietary supplement in fish feed to nonspecifically absorb potentially toxic agents. Activated carbon can be used as an indigestible carrier to prevent the intake of toxic substances through the digestive tract, and thus bamboo charcoal powder is often used as an oral antidote to treat poisoning [15]. Therefore, bamboo charcoal and activated carbon have been widely used as adsorbents or detoxification agents in both animals and humans [16,17]. Previous studies have reported that dietary supplementation with bamboo charcoal has positive effects on animal growth, feed efficiency, specific growth rate, feed intake, nitrogen excretion, and digestion [15,18,19,20,21].

Previous studies on the effects of dietary charcoal supplementation in animals have largely focused on mammals and some fish species, with a particular focus on animal growth [22,23,24,25]. However, to the best of our knowledge, the effects of dietary charcoal supplementation on trace element uptake and antioxidant performance in fish have not been previously characterized.

In previous studies, we determined that juvenile blunt snout bream (*Megalobrama amblycephala*) require a Se dietary intake of 0.958–0.959 mg/kg (our assays were conducted using sodium selenite as a Se source) based on the quadratic fitting method. Nevertheless, the growth performance was significantly affected and the expression of anti-inflammatory factors was inhibited when the dietary Se intake was too high [7]. Given its activity as an insoluble carrier, bamboo charcoal can be used as a dietary supplement in fish feed to absorb and slowly release excess nutrients. However, these hypotheses have not been evaluated in diets containing excessively high Se levels and are thus worth exploring. Therefore, our study sought to investigate the ability of bamboo charcoal to reduce the negative effects of high dietary selenium (Se) concentrations in *Megalobrama amblycephala*.

## 2. Materials and Methods

### 2.1. Ethics Statement

All experimental protocols, methods, and feeding regimes were approved in October 2017 (Authorization NO. 20171013003) by the Institutional Animal Care and Use Committee of the Freshwater Fisheries Research Center, Chinese Academy of Fishery Sciences (Wuxi, China). All fish were anesthetized in well-aerated water with 0.01% tricaine methanesulfonate (MS-222; Sigma, Saint Louis, MO, USA) and sacrificed according to the Care and Use of Laboratory Animals of China guidelines.

### 2.2. Diet Preparation

Five Se-rich experimental diets, marked as the control group, the BC1 group, the BC2 group, the BC3 group, and the BC4 group, were prepared with an average Se content of 1.456 mg/kg and supplemented with 0, 1, 2, 3, and 4 g/kg of bamboo charcoal powder (Table 1). All ingredients were sieved to remove any solid impurities and then thoroughly mixed. Afterward, soybean oil and water were gradually added to produce a sinking pellet (2 mm) using an F-26(II) pelletizer (South China University of Technology, China). Once prepared, the diets were air dried, packed into airtight plastic bags, and stored at −20 °C.

### 2.3. Experimental Procedure

Juvenile blunt snout bream were obtained from the Nanquan farm of the Freshwater Fisheries Research Center (FFCR), Chinese Academy of Fishery Sciences. Prior to the feeding trial, fish were allowed to acclimate for two weeks in indoor circular polyethylene tanks (diameter: 820 mm; height: 700 mm) and fed with a commercial diet containing 33.0% protein and 7.0% lipid (Wuxi Tongwei feedstuffs Co. Ltd., Wuxi, China). Afterward, a total of 400 healthy, similarly sized fish (16.00 ± 0.5 g) were randomly distributed into 20 tanks (20 fish per tank). Each diet was randomly assigned to quadruplicate tanks. The fish were hand-fed carefully four times daily at 8:00, 11:00, 14:00, and 17:00 until apparent satiation (based on visual observation) for 8 weeks. During the feeding trial, the water temperature was kept between 28 and 30 °C, and other parameters were also carefully controlled (dissolved oxygen ≥ 6 mg/L, ammonia nitrogen ≤ 0.2 mg/L, nitrite ≤ 0.2 mg/L, pH 6.8−7.5, natural lighting (12 h light/12 h dark)).

### 2.4. Sample Collection and Analysis

#### 2.4.1. Sample Collection

At the end of the feeding trial, the fish were fasted for 24 h before sampling. All of the fish from each tank were anesthetized with 100 mg/L of tricaine methanesulfonate (MS-222) and weighed to calculate growth performance. Six fish were then randomly collected from each tank. Blood samples were first obtained from the caudal vein using disposable medical syringes with anticoagulant. Plasma was separated and collected by centrifugation at 4000× *g* at 4 °C for 10 min. All samples were stored at −20 °C until required for the analysis of plasma parameters. Liver tissues from each fish were then stripped, flash frozen in liquid nitrogen, and stored at −80°C for downstream analyses of antioxidant enzyme activities and gene expression. Dorsal muscles were also collected to determine nutrient composition.

#### 2.4.2. Laboratory Analysis

Several growth parameters including the weight growth rate (WGR), specific growth rate (SGR), feed conversion ratio (FCR), and protein efficiency ratio (PER) were calculated as follows: WGR = 100 × ((final body weight (g) − initial body weight (g))/initial body weight (g)); SGR = 100 × ((In (final body weight (g)) − In (initial body weight (g)))/days); FCR = dry feed fed (g)/(final body weight (g) − initial body weight (g)); PER = (final body weight (g) − initial body weight (g)/dietary protein consume).

The moisture, crude protein, crude lipid, and ash contents of the experimental diets and fish muscle were analyzed according to the established AOAC (Association of Official Analytical Chemists) guidelines [26]. Selenium concentrations in the diets and fish muscle samples were analyzed via inductively coupled plasma mass spectrometry (ICP-MS) (Institute of Feed Science, Jiangnan University, China).

Several plasma parameters including total protein (TP), albumin (ALB), glucose (GLU), alkaline phosphatase (ALP), total cholesterol (TC), total triglyceride (TG), alanine aminotransferase (ALT), and aspartate aminotransferase (AST) were measured using a Mindray BS-400 automatic biochemical analyzer (Mindray Medical International Ltd., Shenzhen, China). All kits were purchased from Shanghai Zhicheng Biological Co., Ltd. (Shanghai, China).

#### 2.4.3. Determination of Hepatic Antioxidant Parameters

Liver samples were homogenized in ice-cold physiological saline and centrifuged at 4000× *g* for 10 min at 4 °C, after which the hepatic supernatant was collected. Several hepatic antioxidant parameters including catalase (CAT, Kit No. A007-2), total antioxidative capacity (T-AOC, Kit No. A015-1), total superoxide dismutase (T-SOD, Kit No. A001-1), glutathione peroxidase (GSH-Px, Kit No. A005-1), glutathione (GSH, Kit No. A006-1), and malondialdehyde (MDA, Kit No. A003-1) were then measured using commercial kits purchased from Nanjing Jiancheng Bioengineering Institute (Nanjing, China).

#### 2.4.4. Real-Time PCR Analysis

RNA was extracted from livers with the Trizol method according to the instructions of the RNAisoplus kit (Takara, Dalian, China), and RNA quality was detected with the Nano Drop2000 software. RNA samples with OD260 nm/OD280 nm ratios between 1.8 and 2.0 were selected for downstream cDNA reverse transcription. cDNA was synthesized with the PrimeScript^TM^ RT reagent Kit with gDNA Eraser (Takara, Dalian, China). Relative mRNA expression levels were determined via real-time PCR performed using a CFX96 instrument (Bio-Rad, USA) coupled with the One-Step SYBR PrimerScript PLUS RT-PCR Kit (TaKaRa, Dalian, China). Seven genes were selected for qRT-PCR validation, including kelch-like ECH-associated protein 1 (*keap-1*), nuclear factor erythroid 2-related factor 2 (*nrf2*), nuclear factor kappa B (*nfkb*), catalase (*cat*), glutathione peroxidase (*gpx*), manganese superoxide dismutase (*mn-sod*), tumor necrosis factor α (*tnfα*), transforming growth factor α (tgfα), and interleukin 10 (*il10*). β-actin was used as a reference gene (GeneBank accession no. AY170122.2). All primer sequences were published in previous studies (Table 2) and synthesized by Shanghai Sangon Biotech Company (Shanghai, China). Relative expression levels were calculated using the 2^−ΔΔCT^ method.

### 2.5. Statistical Analysis

All data analyses were performed using the SPSS 20.0 software (IBM Corp., Armonk, NY, USA). The data were first tested for normality (Shapiro–Wilk test) and homogeneity of variance (Levene’s test). Differences between the means of the dietary bamboo charcoal powder treatments were identified via one-way analysis of variance (ANOVA) coupled with Tukey’s test; a *p*-value < 0.05 was deemed statistically significant.

## 3. Results

### 3.1. Effects of Se-Rich Dietary Bamboo Charcoal Inclusion Levels on Juvenile Blunt Snout Bream Growth Parameters

The juvenile blunt snout bream analyzed herein exhibited normal feeding rates throughout the 8-week feeding trial, and no mortalities were observed. The growth performance and feed conversion ratio of juvenile blunt snout bream are summarized in Table 3. The WGR and SGR of juvenile blunt snout bream exhibited a strongly significant quadratic relationship with the bamboo charcoal inclusion levels in the Se-rich feed. When the bamboo charcoal content was 3 g/kg, the values of the WGR and SGR reached maximum levels of 177.13% and 1.82%/day, respectively, which were significantly higher than those of the control group and the BC1 group (*p* < 0.05). The FCR had a strongly significant positive linear correlation with the increasing bamboo charcoal powder content in the Se-rich feed (*p* < 0.001). Moreover, the FCRs of the BC3 and BC4 groups were significantly higher than those of the control and BC1 groups (*p* < 0.05). In contrast, the PER was strongly significantly negatively correlated with the increasing bamboo charcoal powder content in the Se-rich feed (*p* < 0.001). The PER values of the BC3 and BC4 groups were significantly lower than those of the control and BC1 groups (*p* < 0.05).

### 3.2. Effects of Se-Rich Dietary Bamboo Charcoal Inclusion Levels on the Muscle Nutrient Profile of Juvenile Blunt Snout Bream

Table 4 summarizes the muscle nutrient profiles of juvenile blunt snout bream. The inclusion levels of bamboo charcoal powder in the Se-rich feed had negligible effects on muscle moisture, crude lipid, and ash contents (*p* > 0.05). When the bamboo charcoal inclusion level was 2 g/kg in the diet, the crude protein in the muscle was significantly lower than that in the BC1 group (*p* < 0.05). The Se content in muscle tissue exhibited a strongly significant negative linear correlation with the bamboo charcoal content in the feed (*p* = 0.000). The Se content in the muscle of the BC4 group was significantly lower than that of the control and BC1 groups (*p* < 0.05).

### 3.3. Effects of Se-Rich Dietary Bamboo Charcoal Inclusion Levels on Plasma Physiological and Biochemical Parameters

Table 5 summarizes the plasma physiological and biochemical parameters of juvenile blunt snout bream. The levels of TP, TC, TG, ALT, and AST were not affected by dietary bamboo charcoal supplementation (*p* > 0.05). Plasma ALB, GLU, and ALP tended to increase first and then decrease with increasing bamboo charcoal powder content, with the highest levels occurring in the BC3, BC3, and BC1 groups, respectively. Moreover, plasma ALB in the BC3 group was significantly higher than that in the BC1 group (*p* < 0.05); the plasma GLU level in the BC2 and BC3 groups was significantly higher than that in the BC4 group (*p* < 0.05). Further, the plasma ALP level in the BC1 group was significantly higher than that in the BC3 and BC4 groups (*p* < 0.05).

### 3.4. Effects of Se-Rich Dietary Bamboo Charcoal Inclusion Levels on Liver Antioxidant Abilities

Table 6 illustrates the hepatic antioxidant parameters of juvenile blunt snout bream fed with different levels of dietary bamboo charcoal supplementation. In general, the presence of 3 g/kg of bamboo charcoal in the feed significantly enhanced the enzyme activities of CAT, T-SOD, T-AOC, and GSH-Px compared to the control group and the other supplementary groups (*p* < 0.05). Further, the activity of GSH-Px was significantly lower than that in the BC2 group (*p* < 0.05). The MDA contents in the control group, BC3 group, and BC4 group decreased and were significantly different from those of the BC1 group (*p* < 0.05). The hepatic GSH contents of different groups were not affected by the dietary bamboo charcoal concentration (*p* > 0.05).

### 3.5. Effects of Se-Rich Dietary Bamboo Charcoal Inclusion Levels on Anti-Inflammatory Gene Expression in Juvenile Blunt Snout Bream Livers

**Figure 1:**
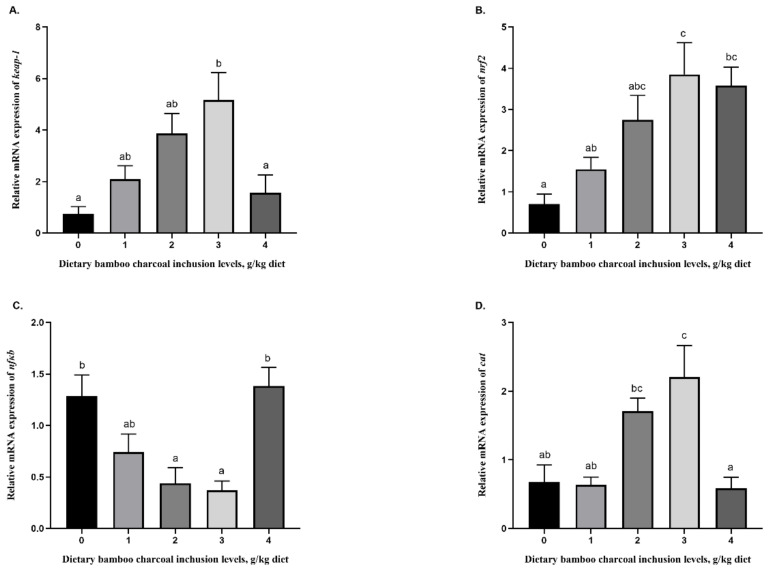

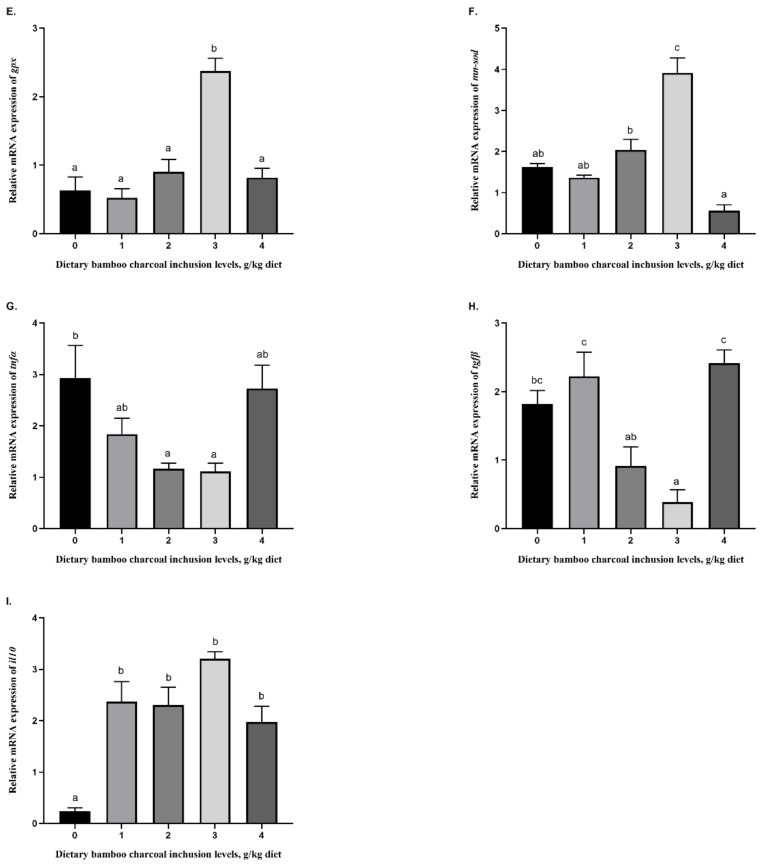
Effects of dietary bamboo charcoal levels on the relative expression of the *keap-1* (**A**), *nrf2* (**B**), *nfkb* (**C**), *cat* (**D**), *gpx* (**E**), *mn-sod* (**F**), *tnfα* (**G**), *tgfβ* (**H**), and *il10* (**I**) genes in blunt snout bream liver. All data are reported as means ± SEM (*n* = 4). a,b,c: Bars labeled with different superscripts are significantly different from each other according to Tukey’s test (*p* < 0.05).

The relative expressions of *keap-1* and *nrf2* exhibited similar trends, with both gradually increasing in the BC3 group and then decreasing. Furthermore, *keap-1* expression was significantly higher than that of the control group and the BC4 group, whereas *nrf2* expression was significantly higher than that of the control group and the BC1 group (Figure 1A,B; *p* < 0.05). In contrast, hepatic *nfkb* expression decreased with increasing dietary bamboo charcoal inclusion levels from 0 to 3 g/kg and then dramatically increased at the 4 g/kg inclusion level, reaching values that were significantly higher than those in the BC2 and BC3 groups (Figure 1C; *p* < 0.05). The expressions of the hepatic genes *cat*, *gpx*, and *mn-sod* were all elevated in the BC3 group. Interestingly, *cat* expression in the BC3 group was higher than that of the control, BC1, and BC4 groups (Figure 1D; *p* < 0.05). Similarly, the expressions of *gpx* and *mn-sod* were significantly higher than those of the other groups (Figure 1E,F; *p* < 0.05). The *tnfα* and *tgfβ* genes were downregulated in groups fed with Se-rich feed supplemented with 0 to 3 g/kg bamboo charcoal but were upregulated in the group supplemented with 4 g/kg of dietary bamboo charcoal powder. The mRNA expression of *tnfα* was the lowest in groups BC2 and BC4 (Figure 1G; *p* < 0.05), whereas the expression of *tgfβ* was the lowest in group BC4 (Figure 1H; *p* < 0.05). Hepatic *il10* was significantly upregulated in the groups fed with bamboo charcoal powder-supplemented diets (1, 2, 3, and 4 g/kg) compared to the control group (Figure 1I; *p* < 0.05).

## 4. Discussion

Many studies have reported that dietary charcoal supplementation promotes fish growth. Thu et al. [23] fed *Paralichthys olivaceus* with diets containing different amounts of bamboo charcoal (0%, 0.25%, 0.5%, 1%, 2%, and 4%) for 50 day. Interestingly, the 0.5% group exhibited the highest WG and SGR values. Similarly, Michael et al. [22] fed red tilapia with diets containing different amounts of charcoal (0, 10, 20, 30, and 40 g/kg) for 60 day. The authors reported that the 30 g/kg and 40 g/kg groups exhibited significantly higher WG and SGR values, whereas the FCR of the 30 g/kg and 40 g/kg groups decreased significantly. This may have been because bamboo charcoal can absorb toxic substances in the intestine, thus improving intestinal function (i.e., nutrient absorption) and promoting growth [15]. Previous studies have demonstrated not only that high supplementation levels of Se in feeds affect its utilization but also that excessive dietary Se is linked to fish growth inhibition (e.g., reduced WGR and SGR, increased mortality) [7,27]. In this study, both the 2 and 3 mg/kg bamboo charcoal concentrations in the Se-rich feed significantly improved the WGR and SGR values of juvenile blunt snout bream, suggesting that bamboo charcoal supplementation improves dietary Se utilization when administered in appropriate amounts. This was likely because bamboo charcoal absorbs excess nutrients in feed and improves the absorption and metabolic capacity of the digestive tract by slowing down the release of nutrients. However, dietary bamboo charcoal supplementation significantly reduces the average daily feed intake [28]. Additionally, carbon supplementation has also been reported to inhibit growth when exposure concentrations are too high [29]. These observations are likely attributed to two factors. First, one of the components of bamboo charcoal powder may have adverse effects on the digestive tract. Second, bamboo charcoal powder may induce bowel movements, thus shortening food retention and nutrient digestion and absorption. In turn, this reduces protein efficiency and increases feeding rates. Likewise, in our study, increasing bamboo charcoal concentrations led to increases in FCR values coupled with decreases in the PER.

Bamboo charcoal has a high absorption capacity and is thus considered an ideal insoluble carrier due to the unique structure of the micropores in the bamboo stem. For example, a study reported that the tolerance of Nile tilapia to waterborne heavy metals increased after feeding the fish with an activated carbon-supplemented feed for 8 weeks, thus reducing the accumulation of heavy metals in fish tissues [30]. Likewise, the accumulation of heavy metals (Pb, Cd, and Cu) was minimized in *Huso huso* muscle tissues after being fed with a diet supplemented with 15 g/kg of activated carbon for 60 days [31]. In our study, selenium accumulation in the muscle tissue of juvenile blunt snout bream decreased with increasing dietary bamboo charcoal content. In contrast, another study reported that dietary supplementation with commercial charcoal had negligible effects on the protein, crude fat, and ash contents of red tilapia muscle tissue [22]. Our study detected that the muscle protein content of blunt snout bream juveniles increased when the bamboo charcoal content in the feed was 2 g/kg and 3 g/kg.

Blood biochemical parameters such as ALB, ALT, AST, and ALP are common indicators of the physiological, liver health, and immune status of fish [13,32,33]. Previous studies have reported that excess dietary Se (1.46 mg/kg) led to significantly lower ALB plasma levels in juvenile blunt snout bream, thus highlighting the negative effects of high Se dietary exposure on the immune response of these organisms [7]. Our results indicate that the presence of bamboo charcoal (3 g/kg) in the Se-rich feed reduced ALP plasma levels and improved liver metabolism and detoxification capacity in juvenile blunt snout bream. Similarly, another study demonstrated that dietary supplementation with 25 g/kg of activated carbon in *Huso huso* resulted in maximum ALB plasma levels and minimum ALT and AST plasma levels during a heavy metal (Pb, Cd, and Cu) stress challenge [31]. This demonstrated that activated carbon can alleviate the negative effects of heavy metals on blood health. ALT, AST, and ALP plasma levels decreased drastically at bamboo charcoal levels of 0.5% and 1% in red tilapia after a 60 day feeding trial [34]. In carp (*Cyprinus carpio* L.), it was reported that the plasma levels of ALT, AST, and ALP were significantly lower when the bamboo charcoal content in the feed was 1–4% [25]. Nevertheless, the optimal level of dietary bamboo charcoal or activated carbon may vary between studies. This can be attributed to species-specific differences, as well as carbon sources, and fish feeding habits.

The presence of 2% bamboo charcoal in diets with high deoxynivalenol concentrations significantly increased the activity of T-AOC and reduced the MDA content [35]. Likewise, dietary supplementation with activated carbon can enhance the tolerance of tilapia to oxidative stress and reduce heavy metal uptake in these organisms [36]. Our previous study detected that the activities of enzymes related to the antioxidant system were inhibited when the Se content in the feed was 1.46 mg/kg diet, suggesting that excessive dietary exposure to Se may chronically impair the functions of the antioxidant system [7]. While improvements in the MDA content were observed at 1 g/kg inclusion levels, upon close inspection, the data suggest that when considering an inclusion level that can produce the maximum beneficial effects on these hepatic antioxidant enzyme activities and MDA levels, and thus on the overall health of fish, an improvement in most parameters (CAT, T-SOD, T-AOC, GSH-Px, and GSH) can be observed at the 3 g/kg inclusion level compared to the control group. Therefore, BC supplementation at 3 g/kg inclusion can be applied in Se-rich diets of blunt snout bream to enhance the hepatic activities.

Nrf2 plays a central role in cell protection against a wide variety of stressors. Many studies have characterized the molecular sequences and regulatory functions of nrf2 in teleosts including grass carp (*Ctenopharyngodon idellus*) [37], zebrafish (*Danio rerio*) [38], *Pseudosciaena crocea* [39], *Cyprinus carpio* var. Jian [40], and *Coilia nasus* [41]. The Nrf2 signaling pathway is activated as a stress compensation mechanism under environmental or nutritional stress. For instance, nrf2 expression was elevated in isoniazid-induced liver injury in zebrafish larvae [42]. Our previous study identified a link between nrf2 upregulation caused by oxidative stress and hyperglycemia in juvenile blunt snout bream (*Megalobrama amblycephala*) [43]. The Nrf2-Keap1 system is an evolutionarily conserved intracellular defense mechanism to counteract oxidative stress and to preserve cellular homeostasis [44,45,46]. In stressful conditions, Nrf2 is activated, detaches from Keap1, and translocates to the nucleus to induce the expression of antioxidant and metabolic genes. Additionally, the Nrf2-Nfκb pathways regulate the physiological homeostasis of the cellular redox status, as well as stress and inflammation responses. Compounds that decrease the inflammatory response by suppressing NF-κB signaling are known to activate the Nrf2 pathway. Consistently, our study detected that 0 to 3 k/kg bamboo charcoal supplementation in Se-rich diets upregulated the mRNA expression of *keap1* and *nrf2*, whereas *nfkb* expression exhibited the opposite trend. It is widely accepted that the activities of antioxidant enzymes could partly result from the induction of their encoding genes to decrease the inflammatory response, which has been reported to be regulated by the Nrf2/Keap1/Nfκb signaling pathway in fish [47,48,49]. In addition to the transcriptional changes of the aforementioned Nrf2/Keap1/Nfκb signaling genes, the gene expressions of the antioxidant enzymes *cat*, *gpx*, and *mn-sod* were consistently enhanced in the group fed with the diet with 3 k/kg bamboo charcoal supplementation. Inflammation is generally a pervasive response to disturbances in tissue homeostasis due to a variety of stimuli, which involves the activation of innate and adaptive immunity. For instance, pro-inflammatory cytokines (e.g., tnfα, tgfβ, IL-1β, and IL-6) and anti-inflammatory cytokines (e.g., il10) have been described to play a critical role in the inflammatory process. Excessive dietary nutrients always lead to adverse effects on fish. For example, pro-inflammatory cytokines were upregulated and anti-inflammatory cytokines were downregulated in grass carp fed with excessive amounts of dietary histidine [50]. Nevertheless, some feed additives could alleviate these adverse effects and even improve immunity. Song et al. [51] described that dietary emodin supplementation upregulated the expression of the antioxidant gene *cat* while inhibiting *Il-1β*, *tnfα,* and *tgfβ* by modulating the Nrf2-Keap1 signaling pathway. In the early life stages of the zebrafish, the antioxidant enzymes SOD, CAT, and GPx were activated due to Nrf2 overexpression, thus preventing perfluorooctanesulfonic acid from inducing oxidative stress [52]. Consistent with these results, the pro-inflammatory cytokines *tnfα* and *tgfβ* were inhibited in the 2–3 g/kg bamboo charcoal supplementation groups in our study, whereas the expression of the anti-inflammatory cytokine *il10* was upregulated in the bamboo charcoal supplementation groups compared with the Se-rich diet control group.

## 5. Conclusions

Considering that charcoal is a nonspecific adsorbent, its application in the diets of food animals may potentially cause the adsorption of not only toxins but also nutrients from the gastrointestinal tract. Therefore, both positive and negative effects on the physiological functions, growth, and overall health of an organism can be expected. Taken together, our results indicate that 2–3 g/kg of bamboo charcoal supplementation in Se-rich feed enhanced the growth performance, physiological status, and antioxidant enzyme activities of blunt snout bream. Moreover, bamboo charcoal supplementation in Se-rich diets promoted the activation of Nrf2-Keap1 signaling and suppressed NF-κB signaling to mobilize the antioxidant system and inhibit the inflammatory response. These results indicate that bamboo charcoal is an effective immunostimulant that could protect organisms from the adverse effects induced by excessive dietary Se intake, thus providing insights into the mechanisms by which excessive nutrient concentrations affect aquaculture productivity.

## Figures and Tables

**Table 1:** Ingredients and nutrient composition of experimental diets.

Ingredients (g/kg)	Dietary Bamboo Charcoal Inclusion Levels				
	0 (Control)	1.0 (BC1)	2.0 (BC2)	3.0 (BC3)	4.0 (BC4)
Bamboo charcoal powder ^1^	0	1	2	3	4
Sodium selenite (mg/kg) ^2^	4	4	4	4	4
Fish meal ^3^	40	40	40	40	40
Soybean meal ^3^	390	390	390	390	390
Rapeseed meal ^3^	150	150	150	150	150
Cottonseed meal ^3^	100	100	100	100	100
Wheat meal ^3^	150	150	150	150	150
Soybean oil	60	60	60	60	60
Vitamin premix ^4^	5	5	5	5	5
Mineral premix (Se-free) ^5^	5	5	5	5	5
Carboxymethyl cellulose	80	79	78	77	76
Monocalcium phosphate	10	10	10	10	10
Vitamin C	10	10	10	10	10
Sum	1000	1000	1000	1000	1000
Nutrition composition, % fresh					
Crude protein	36.2	36.1	36.8	36.0	36.2
Crude lipid	8.8	8.6	8.7	8.7	8.6
Ash	7.8	7.3	7.9	7.8	7.6
Total Se concentration (mg/kg)	1.48	1.46	1.46	1.44	1.44

^1^ Obtained from Shandong Wedo Agribusiness and Technology Co., Ltd. (Shandong, China). ^2^ Obtained from Shandong Ono Chemical Co., Ltd. (Shandong, China), purity is 98%. ^3^ Obtained from Wuxi Tongwei feedstuffs Co., Ltd. (Jiangsu, China). Fish meal, selenite 4.70 mg/kg; soybean meal, selenite 0.13 mg/kg; rapeseed meal, selenite 1.10 mg/kg; cottonseed meal, selenite 0.27 mg/kg. ^4^ Provided by Wuxi Hanove Animal Health Products Co., Ltd. (Wuxi, China). Vitamins (IU/kg or mg/kg of premix): vitamin A, 900,000 IU; vitamin D, 25,000 IU; vitamin E, 4500 mg; vitamin K3, 220 mg; vitamin B1, 320 mg; vitamin B2, 1090 mg; vitamin B5, 2000 mg; vitamin B6, 5000 mg; vitamin B12, 116 mg; pantothenic acid, 1000 mg; folic acid, 165 mg; choline, 60,000 mg; biotin, 50 mg; niacin, 2500 mg. ^5^ Obtained from Shanghai Zhanyun Chemical Reagent Co., Ltd. (Shanghai, China). Mineral premix composition (g/kg of premix): calcium diphosphate, 20 g; sodium chloride, 2.6 g; potassium chloride, 5 g; magnesium sulfate, 2 g; ferrous sulfate, 0.9 g; zinc sulfate, 0.06 g; cupric sulfate, 0.02 g; manganese sulfate, 0.03 g; cobalt chloride, 0.05 g; potassium iodide, 0.004 g.

**Table 2:** Primers used for real-time PCR analysis.

Primer	Sequences	
	Forward Primer (5′−3′)	Reverse Primer (5′−3′)
β-actin	TCGTCCACCGCAAATGCTTCTA	CCGTCACCTTCACCGTTCCAGT
*keap-1*	AATATCCGCCGGCTGTGTAG	TGAGTCCGAGGTGTTTCGTG
*nrf2*	GGGGAAGTCCTTGAACGGAG	AACCAGCGGGAATATCTCGG
*nfκb*	AGTCCGATCCATCCGCACTA	ACTGGAGCCGGTCATTTCAG
*cat*	CAGTGCTCCTGATACCCAGC	TTCTGACACAGACGCTCTCG
*gpx*	GAACGCCCACCCTCTGTTTG	CGATGTCATTCCGGTTCACG
*mn-sod*	AGCTGCACCACAGCAAGCAC	TCCTCCACCATTCGGTGACA
*tnfα*	TGGAGAGTGAACCAGGACCA	AGAGACCTGGCTGTAGACGA
*tgf* *β*	ACTGGACAAACAGAGAGGCG	CAGGGGAGTTGCCGTTAGAG
*il10*	GTGTTTTCGGGTGCAAGTGG	ATGAACGAGATCCTGCGCTT

**Table 3:** Growth performance of blunt snout bream fed with the experimental diets ^1^.

	Dietary Bamboo Charcoal Powder Inclusion Levels, g/kg Diet					*p*-Value	
	0 (Control)	1.0 (BC1)	2.0 (BC2)	3.0 (BC3)	4.0 (BC4)	Linear	Quadratic
Initial weight (g)	16.67 ± 0.06	16.76 ± 0.02	16.76 ± 0.09	16.58 ± 0.10	16.67 ± 0.05	0.439	0.596
Final weight (g)	43.77 ± 0.44 ^a^	44.24 ± 0.39 ^ab^	45.93 ± 0.34 ^b^	45.94 ± 0.65 ^b^	44.45 ± 0.57 ^ab^	0.129	0.010
WGR (%)	162.61 ± 2.20 ^a^	163.93 ± 2.04 ^ab^	174.15 ± 1.40 ^bc^	177.13 ± 3.16 ^c^	166.67 ± 3.16 ^abc^	0.065	0.007
SGR (%/day)	1.72 ± 0.01 ^a^	1.73 ± 0.01 ^ab^	1.80 ± 0.01 ^bc^	1.82 ± 0.02 ^c^	1.75 ± 0.02 ^abc^	0.065	0.007
FCR	1.62 ± 0.01 ^a^	1.68 ± 0.01 ^b^	1.73 ± 0.01 ^bc^	1.74 ± 0.02 ^c^	1.78 ± 0.01 ^c^	<0.001	<0.001
PER	1.69 ± 0.01 ^c^	1.66 ± 0.01 ^bc^	1.61 ± 0.01 ^ab^	1.59 ± 0.02 ^a^	1.57 ± 0.01 ^a^	<0.001	<0.001

^1^ All data are reported as means ± SEM (*n* = 4). ^a,b,c^ Means in the same row with different superscripts are significantly different from each other according to Tukey’s test (*p* < 0.05).

**Table 4:** Muscle composition in juvenile blunt snout bream fed with the experimental diets ^1^.

	Dietary Bamboo Charcoal Powder Inclusion Levels, g/kg Diet					*p*-Value	
	0 (Control)	1.0 (BC1)	2.0 (BC2)	3.0 (BC3)	4.0 (BC4)	Linear	Quadratic
Moisture (g/kg)	766.84 ± 1.83	762.44 ± 1.97	763.94 ± 1.64	759.37 ± 3.26	766.08 ± 2.70	0.451	0.160
Crude protein (g/kg)	185.66 ± 1.68 ^ab^	189.95 ± 1.59 ^b^	182.91 ± 1.24 ^a^	187.03 ± 1.26 ^ab^	188.05 ± 1.93 ^ab^	0.666	0.695
Crude lipid (g/kg)	31.48 ± 1.17	30.55 ± 2.43	30.75 ± 1.84	29.81 ± 1.94	29.85 ± 2.08	0.505	0.797
Ash (g/kg)	20.83± 0.79	20.59 ± 0.81	18.36 ± 0.51	19.86± 0.46	20.73 ± 0.59	0.656	0.061
Se (mg/kg)	0.57± 0.03 ^c^	0.54 ± 0.03 ^bc^	0.49 ± 0.01 ^abc^	0.46 ±0.01 ^ab^	0.42 ± 0.03 ^a^	<0.001	<0.001

^1^ All data are reported as means ± SEM (*n* = 4). ^a,b,c^ Means in the same row with different superscripts are significantly different from each other according to Tukey’s test (*p* < 0.05).

**Table 5:** Plasma biochemical parameters of blunt snout bream fed with the experimental diets ^1^.

	Dietary Bamboo Charcoal Powder Inclusion Levels, g/kg Diet					*p*-Value	
	0 (Control)	1.0 (BC1)	2.0 (BC2)	3.0 (BC3)	4.0 (BC4)	Linear	Quadratic
ALB (g/L)	13.61 ± 0.48 ^ab^	12.25 ± 0.51 ^a^	14.18 ± 0.66 ^ab^	14.85 ± 0.60 ^b^	13.52 ± 0.48 ^ab^	0.206	0.370
GLU (mmol/L)	9.62 ± 0.32 ^ab^	9.89 ± 0.65 ^ab^	11.62 ± 0.74 ^b^	11.95 ± 0.65 ^b^	8.93 ± 0.62 ^a^	0.665	0.006
ALP (U/L)	37.88 ± 3.05 ^ab^	49.01 ± 4.53 ^b^	38.67 ± 4.15 ^ab^	32.55 ± 2.30 ^a^	33.89 ± 3.24 ^a^	0.031	0.059
TP (g/L)	31.99 ± 0.91	32.91 ± 1.77	32.75 ± 1.19	33.15 ± 1.09	30.95 ± 1.35	0.685	0.463
TC (mmol/L)	6.80 ± 0.14	7.66 ± 0.39	7.26 ± 0.34	6.99 ± 0.30	6.95± 0.29	0.647	0.311
TG (mmol/L)	2.83 ± 0.06	2.80 ± 0.17	2.79 ± 0.13	3.06 ± 0.12	2.65 ± 0.016	0.962	0.536
ALT (U/L)	7.54 ± 1.03	8.83 ± 0.88	6.12 ± 0.50	6.33 ± 0.69	6.85 ± 0.95	0.159	0.338
AST (U/L)	259.58 ± 10.75	256.08 ± 24.10	275.11 ± 26.86	217.9 ± 22.59	218.15 ± 21.10	0.090	0.185

^1^ All data are reported as means ± SEM (*n* = 4). ^a,b^ Means in the same row with different superscripts are significantly different from each other according to Tukey’s test (*p* < 0.05). ALB, albumin; GLU, glucose; ALP, alkaline phosphatase; TP, total protein; TC, total cholesterol; TG, total triglyceride; ALT, alanine aminotransferase; AST, aspartate aminotransferase.

**Table 6:** Hepatic antioxidant enzymes of blunt snout bream fed with the experimental diets ^1^.

	Dietary Bamboo Charcoal Powder Inclusion Levels, g/kg Diet					*p*-Value	
	0 (Control)	1.0(BC1)	2.0(BC2)	3.0(BC3)	4.0(BC4)	Linear	Quadratic
CAT (U/mgprot)	11.68 ± 0.94 ^a^	15.52 ± 1.70 ^a^	16.07 ± 1.47 ^a^	22.94 ± 1.51 ^b^	14.57 ± 1.33 ^a^	0.009	0.001
T-SOD (U/mgprot)	60.84 ± 3.01 ^a^	52.87 ± 5.19 ^a^	57.12 ± 5.02 ^a^	81.38 ± 5.05 ^b^	61.95 ± 3.95 ^a^	0.037	0.114
T-AOC (U/mgprot)	0.14 ± 0.02 ^a^	0.46 ± 0.09 ^a^	0.45 ± 0.09 ^a^	0.98 ± 0.10 ^b^	0.44 ± 0.09 ^a^	0.002	<0.001
GSH-Px (U/mgprot)	88.76 ± 6.07 ^ab^	85.50 ± 7.50 ^a^	129.87 ± 11.55 ^b^	180.76 ± 10.77 ^c^	120.79 ± 6.23 ^ab^	0.002	0.002
GSH (mg/gprot)	4.43 ± 0.64	5.21 ± 1.00	3.60 ± 0.74	3.54 ± 0.47	5.19 ± 0.87	0.911	0.501
MDA (nmol/mgprot)	7.08 ± 0.47 ^a^	11.62 ± 0.86 ^b^	9.18 ± 0.85 ^ab^	7.08 ± 0.57 ^a^	7.96 ± 0.61 ^a^	0.311	0.028

^1^ All data are reported as means ± SEM (*n* = 4). ^a,b,c^ Means in the same row with different superscripts are significantly different from each other according to Tukey’s test (*p* < 0.05). CAT, catalase; T-SOD, total superoxide dismutase; T-AOC, total antioxidative capacity; GSH-Px, glutathione peroxidase; GSH, glutathione; MDA, malondialdehyde.

## Data Availability

The data presented in this study are contained within the article.
